# A survey of veterinary professionals in Sweden: Adverse event reporting and access to product safety information

**DOI:** 10.1002/vro2.18

**Published:** 2021-08-05

**Authors:** James Mount, Karin Sjöström, Veronica Arthurson, Sanna Kreuger

**Affiliations:** ^1^ Department of Drug Safety, Veterinary Medicine Group Swedish Medical Products Agency (Läkemedelsverket) Uppsala Sweden

## Abstract

**Background:**

Pharmacovigilance based on spontaneously reported suspected adverse events (AEs) from veterinary professionals is a powerful tool for detecting potential risks of using medicinal products. However, it is heavily dependent on the voluntary participation of veterinary professionals. Estimates suggest that over 90% of suspected AEs remain unreported. This survey was conducted to accumulate information on current practices and attitudes of Swedish veterinary professionals in relation to AE reporting and their perceptions of the accessibility of updated product safety information.

**Methods:**

Swedish veterinary professionals were surveyed using a web‐based questionnaire prepared by the Swedish Medical Products Agency (SMPA). The survey included three sections with 13 questions and was distributed via several communication channels, including the Swedish Veterinary Association.

**Results:**

The survey was answered by 412 veterinary professionals, including veterinarians and licensed veterinary nurses. The survey identified that most veterinarians comply with national legislation by reporting directly to the SMPA, but not all observed AEs are reported. Veterinary professionals indicated that it is important to have an easy and efficient reporting system, preferably directly from an electronic medical records system. Feedback is considered important. Veterinary nursing staff could potentially improve the reporting rate of suspected AEs in Sweden. The degree of knowledge relating to the reporting of AEs varies among professionals, thus impacting on reporting frequency. A single source of product safety information is mainly used, and improvements are required to enhance accessibility and distribution of updated product safety information.

**Conclusions:**

The insight gained from this survey will be used to influence attitudes and facilitate adaptations needed to fulfil the requirements of the European Union regulations. To reduce underreporting of AEs and facilitate access to updated product safety information, various approaches are required including educational interventions, new digital reporting tools and adaption of communication strategies.

## INTRODUCTION

Reporting of suspected adverse events (AEs) is fundamental to identify and minimise the risks of using veterinary or human medicinal products in animals. However, reporting is heavily dependent on the voluntary participation and motivation of veterinary professionals to report any AEs. Under‐reporting of AEs is documented among veterinary professionals and estimates suggest that approximately 90% of AEs are unreported.^1^, ^2^, ^3^ An AE is defined as any unfavourable and unintended reaction in any animal to a veterinary (including off‐label use) or human medicinal product. This definition also includes reports of suspected lack of efficacy, environmental incidents, human exposure, transmission of an infectious agent and exceeding maximum residue limits.^4^

Authorisation and registration of veterinary medicinal products (VMPs) is preceded by extensive pharmacological and toxicological studies. However, only the most common AEs are identified during pre‐clinical and clinical studies. Uncommon AEs, interactions, AEs with delayed onset, batch related issues or specific events that arise in certain species or breeds, are often only detected when the product is used more extensively in clinical practice. In clinical practice, products are used for a boarder range of indications and clinical situations (including other breeds and categories of animals) compared to the regulated conditions of clinical trials, which can give rise to a different profile of AEs. Therefore, it is important to monitor VMPs throughout the lifetime of a product and specifically to closely monitor newly authorised products during the first years of their use.

In Sweden, veterinarians have a legal obligation to report all serious and unforeseen AEs following the use of a veterinary or human medicinal product in animals to the Swedish Medical Products Agency (SMPA). This includes cases of human exposure.^5^ AE reports are collated, together with reports from other national competent authorities (NCAs) and marketing authorisation holders (MAHs) into a single European database, EudraVigilance Veterinary, at the European Medicines Agency (EMA).^6^ As of June 2021, this database contained around 473,000 reports of AEs. These reports ultimately contribute to the collective effort within the European Union (EU) to continuously monitor and evaluate AEs when using veterinary and human medical products in animals, known as veterinary pharmacovigilance.

Veterinary pharmacovigilance is defined as the science and activities relating to the detection, assessment, understanding and prevention of AEs or any other problem related to a medicinal product.^4^ There are two principle objectives. The first objective is to identify suspected AEs following the use of veterinary or human medicines in animals and to continuously monitor and assess these events to identify any potential risks or safety issues. The second objective is to communicate any identified risks or safety issues to the end user in a timely manner. The aim is to ensure that the benefits of using a medicinal product continue to outweigh the risks.

An EU Directive has, for several decades, outlined requirements and responsibilities of reporters, NCAs and MAHs during these pharmacovigilance activities.^7^ In 2018, a revision was undertaken and a new EU Regulation on VMPs will come into effect on 28 January 2022.^4^ Revisions focus on improving the efficiency of detecting potential risks, reducing administrative burden and improving communication. In the context of pharmacovigilance, communication relates to informing end users about relevant changes to product safety information in a timely manner.

Previous surveys on AE reporting have been conducted with the primary focus on veterinarians at a European level or within other member countries.^1^, ^2^, ^3^ This web‐based survey was conducted in order to gather information from all categories of veterinary professionals in Sweden. The primary aim of this survey was to gain insight into AE reporting practices of Swedish veterinary professionals and their perceptions on the accessibility of updated product safety information. Information gathered will form the basis for future developments and improvements to AE reporting, influence attitudes and practices of Swedish veterinary professionals, enhance communication to end users and facilitate the implementation of the new EU regulation at a national level.

## MATERIALS AND METHODS

A web‐based survey (SurveyMonkey) was prepared by the SMPA. The survey was open between 19 October 2020 and 31 December 2020. Distribution to veterinary professionals was achieved via digital newsletters from the SMPA and the Swedish Veterinary Association, a digital exhibit at the annual Swedish Congress of Veterinary Medicine, an article in *VeterinärMagazinet* (an online magazine for veterinary professionals in Sweden) and via Facebook and LinkedIn. The survey was voluntary, anonymous and divided into three sections with 13 questions (Supporting Information‐1). Not all questions were mandatory and free‐text fields were included in the survey. Descriptive statistical analysis was performed using Microsoft Excel (v16.0).

## RESULTS

**FIGURE 1:**
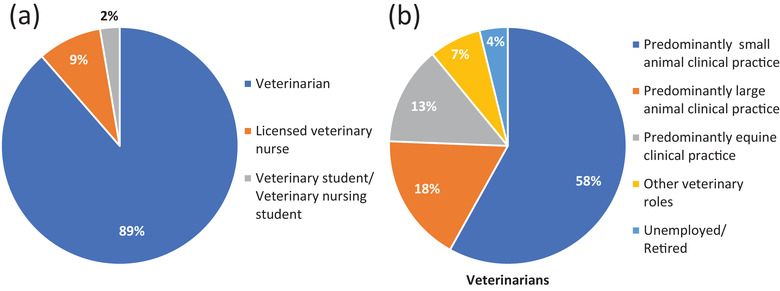
Demography of surveyed veterinary professionals in Sweden. (a) Division of veterinary professionals according to qualification. (b) Division of veterinarians according to field of work. ‘Other veterinary roles’ includes official veterinarians and veterinarians working in zoos, academia, research or animal welfare (*n *= 412)

There were 412 veterinary professionals that completed the survey. The majority of participants (89%) were veterinarians (Figure 1a). This equates to approximately 13% (336 of 2652) of clinically active veterinarians in Sweden, based on estimates derived by the Swedish Board of Agriculture.^8^ Among veterinarians, the majority (58%) worked in predominately small animal practice. The division of other areas of work is displayed in Figure 1b. Licensed Veterinary Nurses (LVs) contributed to 9% of total responses (Figure 1a) and most (94%) worked in small animal practice (data not shown). Veterinary students (including nurses) contributed to the remaining 2% of total responses (Figure 1a).

## CURRENT REPORTING PRACTICES OF VETERINARY PROFESSIONALS IN SWEDEN

**FIGURE 2:**
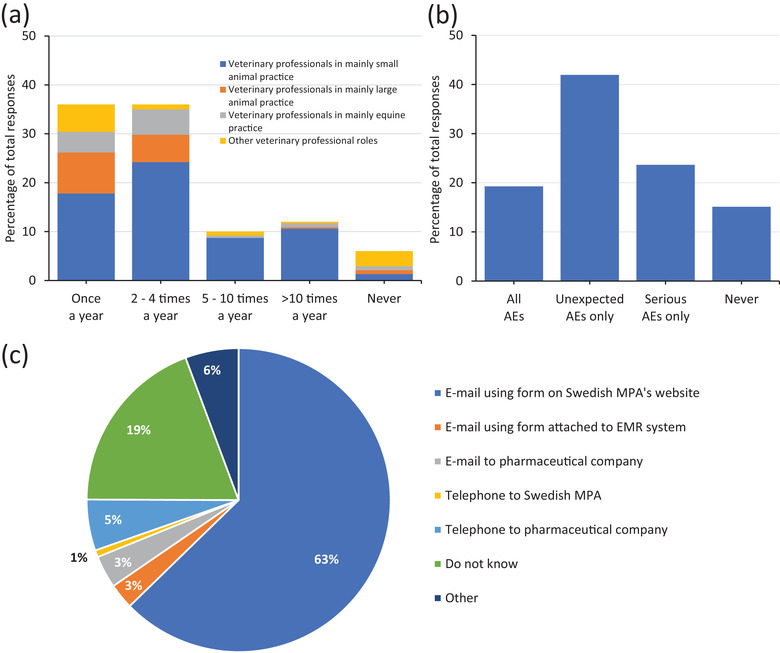
Current practices related to adverse events (AE) reporting of veterinary professionals in Sweden. (a) The number of AEs reportedly submitted annually by veterinary professionals. The distribution of responses for the main categories of clinical practice are shown within each annual reporting frequency (*n *= 409). (b) Types of AE reports currently submitted by veterinary professionals (*n *= 410). (c) Current AE reporting methods used by veterinary professionals (*n *= 405). MPA: Medical Product Agency; EMR: Electronic medical record

First, participants were asked how frequently they suspected or noticed an AE following the use of a medicinal product in an animal. Most participants stated that they suspected or noticed an AE either once per year (36%) or between two and four times a year (36%). The distribution of all responses and the distribution of responses within the main categories of clinical practice are shown in Figure 2a. Our data indicates that veterinary professionals in small animal practice tend to contribute more to the higher annual reporting frequencies.

Second, participants were asked about the type of AEs that are usually reported. A total of 66% of participants stated that they report exclusively unexpected or serious AEs (Figure 2b), whereas 19% of participants stated that they usually reported all AEs with 15% never reporting. The distribution of the responses is shown in Figure 2b.

The third question collected the current method used by veterinary professionals to report AEs. Most participants (63%) stated that they used the reporting form on the SMPAs website. The division of the remaining responses is displayed in Figure 2c. For 19% of participants, they stated that they were unaware of how to report AEs. Of the participants that selected ‘other’, half of free text comments were from LVNs stating that they mainly reported via a veterinarian and, in some instances, reporting was not completed. The other half related to participants stating that they usually submitted a report in the post.

## IMPROVEMENT AND DEVELOPMENT OF ADVERSE EVENT REPORTING

**FIGURE 3:**
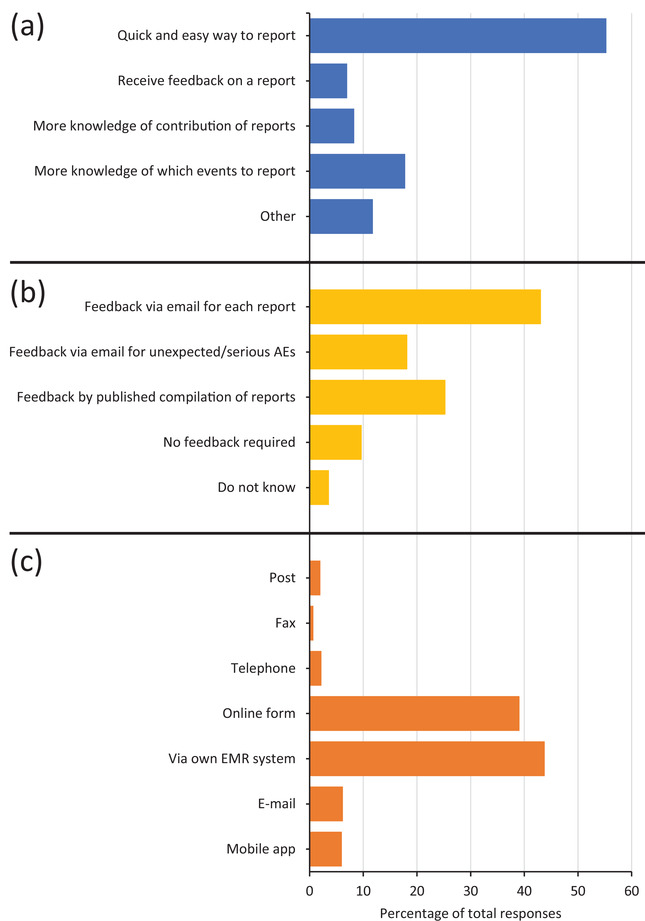
Improvements in adverse events (AE) reporting and feedback suggested by veterinary professionals. (a) Main requirements considered important by veterinary professionals to encourage AE reporting (*n *= 400). (b) Feedback options preferred by veterinary professionals following AE reporting (*n *= 411). (c) Preferred methods of reporting AEs (*n *= 402). Multiple options could be selected by respondents but only the first choice of participants is shown. EMR: Electronic medical record

The next part of the survey focused on gathering of opinions relating to the improvement and development of AE reporting in Sweden. Participants were asked what they considered important and would motivate them to report more frequently. Most participants (55%) considered the availability of a quick and easy method of reporting as the most important factor (Figure 3a). Additional responses are in Figure 3a. Most of “other” responses related to respondents wishing to select a combination of options. Additional comments related to veterinarians highlighting that they have extreme time constraints and a strong tendency to prioritise other tasks. Comments from LVNs related to knowledge of a significant number of unreported AEs within their clinical practices.

When asked if participants would like feedback following submission of an AE report, 43% indicated that they would appreciate feedback on each submitted report (Figure 3b). Feedback refers to a scientific assessment of the AE(s) and/or any actions taken. Other participants indicated that feedback would be considered necessary for unexpected or serious AEs (18%). The distribution of the remaining responses is shown in Figure 3b.

Veterinary professionals were also asked for their preferred method for AE reporting. Most participants preferred to report via an electronic medical records (EMR) system (44%) and via an online form (39%; Figure 3c). The distribution of the remaining 17% of responses is shown in Figure 3c.

## ACCESS TO UPDATED PRODUCT SAFETY INFORMATION

**FIGURE 4:**
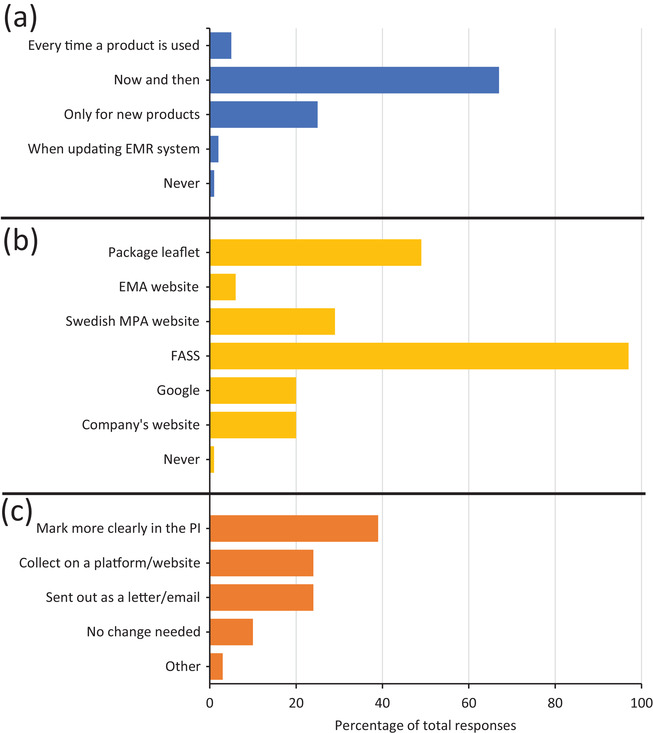
Behaviours and attitudes of veterinary professionals towards the accessibility of updated product safety information. (a) The frequency that veterinary professionals actively search for updated product safety information (*n *= 411). (b) The source of updated product safety information used by veterinary professionals. Respondents could select more than one source and an accumulation of responses is shown (*n *= 412). EMA: European Medicines Agency; MPA: Medical Products Agency. FASS: an online compilation of product information provided by the Swedish pharmaceutical industry association. (c) Improvements considered important by veterinary professionals to facilitate the access of updated product safety information (*n *= 411). PI: Product information

The final section of the survey focused on collecting the attitudes and perceptions towards updated product safety information. Participants were asked how often they actively searched for updated product safety information. Sixty‐seven percent of participants indicated that they periodically search for updated information. For 5% they indicated that they updated themselves every time they used a product. The division of the remaining 28% of responses is displayed in Figure 4a.

Participants were asked to indicate which sources of product safety information that they used. Multiple sources could be selected. A large proportion of respondents (97%) stated that they consulted FASS for updated information (Figure 4b). FASS is an online compilation of product information provided by the Swedish pharmaceutical industry association.^9^ For 29% of respondents indicated that they used the product information on the SMPAs website. Figure 4b shows other sources used by veterinary professionals.

When asked to identify areas of improvement relating to updated product safety information, 39% of participants indicated that updated product safety information should be marked more clearly in the product information (Figure 4c), while 10% expressed the opinion that no changes are required. Figure 4c shows the distribution of all responses. Of the respondents that selected ‘other’, most comments related to participants wishing to select a combination of options. Some comments related to veterinarians emphasizing a need to improve FASS. Other comments suggested that updated product safety information should be made available in the electronic prescribing system via the Swedish eHealth Agency.

**FIGURE 5:**
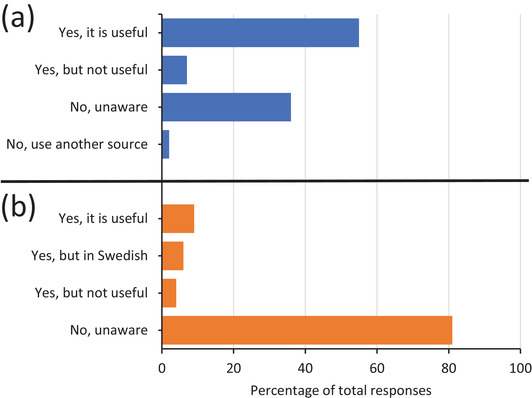
Perceptions and opinions of veterinary professionals towards specific publications focusing on the distribution of updated product safety information. (a) Division of responses to a question related to whether Swedish veterinary professionals were aware of updated product safety information published in the newsletter generated by the Swedish Medicinal Products Agency (*n *= 412). (b) Division of responses to a question related to whether Swedish veterinary professionals were aware of the monthly publication on the European Medicines Agency website regarding Pharmacovigilance regulatory recommendations for centrally authorised veterinary medicinal products (*n *= 409)

The final two questions of the survey focused on specific publications which distribute updated product safety information to veterinary professionals. First, participants were asked whether they were aware of the monthly newsletter from the SMPA; containing updated product safety information for all approved veterinary products.^10^ The majority (55%) of participants stated that they were aware of this newsletter and consider the information useful (Figure 5a). While, 36% stated that they were unaware of the newsletter. Other responses are shown in Figure 5a. Second, veterinary professionals were asked whether they were aware of the monthly publication from EMA.^11^ This publication presents the most recent recommendations for changes to product safety information for centrally approved veterinary products. For 81% of participants they stated that they were unaware of this publication and 9% of participants were aware of and considered the publication useful (Figure 5B).

At the end of the questionnaire, respondents were given the opportunity to state additional comments and suggestions for improvements. Following a review of the 37 comments that were received, the most common related to veterinary professionals suggesting that updated product safety information should be published separately from other information (11 comments), LVNs requesting to receive more focused information (nine comments) and veterinarians emphasizing the need for a quick and easy method to report, given time constraints and highlighting that the current reporting method was time consuming (eight comments).

## DISCUSSION

This survey was developed to gain insight into AE reporting practices of Swedish veterinary professionals and their perceptions on the accessibility of updated product safety information. The aim is to use this data when implementing new strategies to improve reporting rates and enhance communication to end users in Sweden. The main target population was clinically active veterinarians in Sweden with circa 13% participating.^8^ Comparison of the demography of veterinarians in this survey with a survey conducted by the Federation of Veterinarians in Europe indicates a similar distribution of veterinarians among areas of work.^12^ Together, this suggests that the results give a reasonable indication of the current situation. In comparison, 3.1% of veterinarians participated in a survey of veterinarians in Europe.^1^ Although there were fewer responses from LVN and students, which limits the ability to draw general conclusions, their input still helped to identify some important areas to direct future improvements.

Limitations of this study include the non‐random sampling of respondents. In addition, an element of bias could be introduced as the respondents with a greater interest in AE reporting may have a greater motivation to participate in the study, resulting in overestimations. Accuracy of the responses from participants (recall bias) could also impact some of the data collected. The available literature and knowledge related to the attitudes of veterinary professional to AE reporting and factors that influence their practices is limited. However, veterinary pharmacovigilance has many similarities to human pharmacovigilance activities as both regulatory systems share similar goals and obstacles, particularly in relation to spontaneous AE reporting, which allows for comparison.

## CURRENT REPORTING PRACTICES AND INFLUENCING FACTORS

In Sweden and in some other EU countries, veterinarians have a legal obligation to report AEs to their NCA.^5^ Our survey identified that the majority of responding veterinary professionals report AEs and predominately use the reporting form on the SMPAs website. However, our results indicate that there is a significant under‐reporting among veterinary professionals with only a small proportion of participants (19%) stating that they report all suspected AEs and almost 20% stating that they never report. Under‐reporting is not unique to Sweden and has been documented by other surveys within Europe.^1^, ^2^, ^3^ Under‐reporting of AEs is also well recognised within human medicine but the magnitude is difficult to quantify; although estimated to be between 86–94%.^13^, ^14^ Under‐reporting has been suggested to be around 90% in veterinary medicine.^1^, ^3^ Furthermore, figures from EMA indicate that a large proportion of under‐reporting may be related to the use of VMPs in food producing species.^15^ It was observed in our survey that when looking at annual reporting frequencies of veterinary professionals from different fields of work, veterinary professionals in small animal practice dominated the contribution to the higher annual reporting frequencies (i.e., 5–10 times or > 10 times per year) compared with veterinary professionals in large animal practice. Furthermore, veterinary professionals in small animal practice contribute to over 80% of all annual AE reports in Sweden.^16^ Under‐reporting may be a factor in these observations.

In human medicine, the causes of under‐reporting are multifactorial, including lack of time, uncertainty of a causal relationship, lack of awareness of the requirement to report, difficulty accessing reporting forms, lack of understanding the reason for reporting, considering it unnecessary to report known events and believing that a single report will not make any difference.^13^, ^17^ The results from our survey suggest that veterinary professionals share similar reasons for not reporting; the main reason being a lack of time due to high workload. Lack of knowledge, in relation to both the type of events to report and the overall contribution of individual reports, was considered as a potential inhibitor for not reporting more frequently. These responses have identified two specific areas to focus efforts; improving the reporting methods and establishing educational interventions to increase knowledge and awareness.

While it may be considered unrealistic to require the reporting of all suspected AEs, incoming EU Regulations, at the time of writing, will require the reporting of all AEs and not provide scope to limit reporting to certain categories of AEs for example serious and unknown AEs, which is in contrast to the current national legislation; based on EU Directive.^4^, ^5^, ^7^ The new EU Regulation will facilitate decision making for veterinary professionals when reporting AEs but will pose demands on the development of simple and efficient reporting methods. It is important to appreciate that MAHs together with NCAs are required to monitor the frequency and incidence of known AEs, in addition to the identification of any previously unknown AEs and safety issues. The primary goal is to significantly increase the current reporting rate and thus provide more data to efficiently detect potential risks.

It is noteworthy that there is a disparity in responses when comparing the number of participants stating that they do not know how to report (19%) with participants stating that they never report (15%). This could be partly related to the difference in total number of responses received for each question (410 versus 405) or related to a misunderstanding of the questions.

## STRATEGIES FOR IMPROVING ADVERSE EVENT REPORTING

The fundamental aspect of veterinary pharmacovigilance is the retrieval of suspected AEs through spontaneous reports from primarily veterinary professionals, although animal owners and pharmacists may also play a role. It has been repeatedly recognized that continuous improvements to reporting methods are necessary in order to increase spontaneous reporting.^1^ Veterinary professionals who participated in this survey indicated that they have an extreme workload and administrative burden, thus it is particularly important to have a quick and easy method to report AEs. Participants have indicated that the current reporting method available is time consuming. The time that it takes to report AEs across Europe has been shown to vary but in some instances can take over 30 min.^1^ When asked to specify a preferred reporting method, the majority of respondents indicated a digital method; either directly from an EMR system or via an online reporting form. Both reporting methods would reduce the time to report by enabling auto‐population of some information in a report. By contrast, a survey of veterinarians in Germany reported that EMR reporting was not preferred which highlights the importance of adapting approaches at a national level.^2^

The practical challenges of reporting via veterinary EMR systems have been previously highlighted.^1^ The Small Animal Veterinary Surveillance Network (SAVSNET) at the University of Liverpool, UK has developed a solution with the Veterinary Medicines Directorate to enable veterinary practices to submit AE reports from their EMR systems.^18^, ^19^, ^20^ There are currently 250 veterinary practices (around 450–500 individual sites) participating in a pilot study in the United Kingdom. This system is limited to participating practices; this saves time and facilitates reporting. In Sweden, the focus has been mainly on the reporting of AEs from human healthcare EMR systems. The SMPA has developed a system to allow providers of EMR systems to submit reports digitally via a secure server.^21^ This system is reliant on the providers of the EMR systems building reporting forms and enabling auto‐population with information from medical records. Anecdotally, reporting times can be reduced to as little as five minutes in some cases using this approach. Discussions and developments are currently ongoing to replicate this solution for veterinary professionals in Sweden. However, it is important that users encourage providers to develop reporting solutions within their medical record systems.

An online reporting form has only been available for human medicine in Sweden and has been greatly appreciated by human health care professionals and consumers. As a result of the demand indicated by participants in this survey, an online reporting form for veterinary professionals in Sweden will be launched during 2021. Nevertheless, it has been previously noted that the full potential of any new reporting methods is only achieved through continuous promotion and increasing awareness of AE reporting.^15^, ^22^

## ENCOURAGING SPONTANEOUS ADVERESE EVENT REPORTING

It has previously been highlighted that the best way to motivate veterinary professionals is to demonstrate that the reports that are submitted are useful and contribute to the monitoring of medicinal products.^1^ Feedback following submission of reports and educational interventions play a key role.^15^ Veterinary professionals in this survey have indicated that feedback is appreciated, could encourage reporting and is preferred at the level of each report. Traditionally, the SMPA has focused on providing feedback through annual publications similar to other NCAs.^23^, ^24^, ^25^, ^26^ Publication of AEs in human medicine is considered an excellent means of encouraging further reporting.^27^ In 2019, EMA extended the public access to the European database containing AEs following administration of VMPs.^28^ This was an attempt to increase transparency of pharmacovigilance activities and enable veterinary professionals to search for products and related AEs. Ultimately, the degree of feedback will be determined by available resources at national and EU levels; however, personalised feedback will always be more effective and enable the maintenance of good relationships with veterinary professionals.

Educational interventions both at the undergraduate and postgraduate level are considered useful to encourage and increase reporting in human medicine.^13^, ^29^, ^30^, ^31^ Education of veterinary and nursing students has become a particular focus area of improvement within Sweden in order to reduce the lack of knowledge stated by some participants in this survey and increase awareness. A multimodal approach to feedback and educational interventions is considered appropriate for Swedish veterinary professionals.

## THE ROLE OF VETERINARY NURSING STAFF

Although veterinarians have a legal obligation to report AEs in Sweden, any category of the veterinary professional are invited to submit reports. Despite the relatively low participation of LVNs in this survey, a good insight has nevertheless been provided, particularly through a review of their comments. Results indicate that LVNs tend to report via a veterinarian but, in some instances, reporting is not completed. Some LVNs revealed that they were aware of a significant number of AEs in their practices which have gone unreported by veterinarians. This would suggest that LVNs may be in a good position to monitor and retrieve information regarding AEs and potentially have more time and motivation to report.

LVNs play many important roles in clinical veterinary practice and often have much more contact time with animals and their owners. In human medicine, studies have shown that AE reporting by nursing staff can significantly improve the reporting rate and reports are of good quality.^30^, ^32^, ^33^, ^34^ The main factors shown to influence the reporting of nursing staff in human medicine include lack of knowledge, workplace pressures and relationships between nurses and physicians.^33^ The insight from our survey together with evidence from human medicine provide a good reason to increase the involvement of veterinary nursing staff in AE reporting. According to the EU Directive and Regulation, it is possible for NCAs to impose specific requirements on other healthcare professionals in respect of AE reporting which could be a way to increase engagement and motivation of veterinary nursing staff.^4^, ^7^

## ACCESSIBILITY OF UPDATED PRODUCT SAFETY INFORMATION

Following the identification of safety issues or risks, the goal is to inform the end users as quickly as possible to maintain animal and human safety. Safety issue and risks can include changes to the benefit‐risk balance and emergence of AEs or safety precautions. Most issues or risks are communicated to the end user through updating of the product information; regulated at both national and EU levels. Most veterinary professionals in this survey indicate that they periodically search for updated product safety information and tend to use one primary source (FASS).^9^ It was considered important by respondents that updates should be clearly marked and should be distributed to end users either directly or via a specific platform. Improvements to communication strategies is a particular focus of the new EU Regulation and EMA has previously highlighted the importance of communication.^4^, ^15^ Some NCAs have developed solutions to highlight update information.^35^, ^36^ However, a European approach would be more effective, facilitate harmonisation and could be potentially achieved through the future initiative to develop electronic product information (ePI).^37^

Some efforts have already been made at both a national and EU level to facilitate the accessibility of updated safety to end users. In 2018, the SMPA began to publish new updated product safety information in a newsletter directed towards veterinarians; with 1418 subscribers.^10^ Over half of the participants in this survey indicated that this information was useful but improvements or other approaches need to be further investigated at a national level. Distribution of information to other veterinary professionals, including nursing staff, is one area of improvement which was identified from this survey.

The EMA has traditionally published a collation of updated product safety information for centrally authorised VMPs in an annual bulletin with more recent efforts to publish this information continuously throughout the year.^11^, ^38^ In Sweden, centrally authorized VMPs account for around 40% of all registered VMPs. Our results suggest that the awareness of this information provided by the EMA is rather low and highlights an area which requires attention. Collectively this evidence indicates that communication strategies must be adaptable and requires a collaborative effort between MAHs and NCAs, both at a national and EU level, but it is also important to continuously liaise with veterinary professionals. In the future, public access to the comprehensive Union Product Database (UPD) may be a useful tool to increase and facilitate the accessibility of updated product safety information. The UPD (https://www.ema.europa.eu/en/veterinary‐regulatory/overview/veterinary‐medicines‐regulation/union‐product‐database) is due be implemented in 2022 and provide a single source of information on all authorised veterinary medicines within the EU.

In conclusion, several areas were identified which warrant focus and improvement both in Sweden and Europe. To reduce under‐reporting of suspected AEs and facilitate access to updated product safety information, a multi‐strategy approach is required including educational interventions for undergraduates (including veterinary nursing students), development of digital reporting tools and development of communication strategies for knowledge transfer regarding AEs and updated product safety information. Communication is considered the fundamental key to success. The insight gained from this survey will help to improve attitudes to AE reporting in Sweden and facilitate adaptions needed to fulfil the requirements of the new EU regulation. The fundamental aims are to reduce the under‐reporting rate in order to increase the amount of available data and thus be able to efficiently detect possible risks and communicate these to the end user. This will in turn facilitate the provision of safer and more effective veterinary and human medicinal products; ultimately improving animal health, welfare and public health.

## CONFLICT OF INTEREST

The authors declare no conflict of interest.

## Supporting information

SUPPORTING INFORMATION
