# Supplementary material for: A survey of veterinary professionals in Sweden: Adverse event reporting and access to product safety information
Source: Vet Rec Open. 2021 Aug 5;8(1):e18. doi: 10.1002/vro2.18 (PMC8342559; doi:10.1002/vro2.18)
Supplement: Supplementary file 1 — SUPPORTING INFORMATION [file VRO2-8-e18-s001.docx]

# Supporting Information 1 Survey questions

# NB: English translation on pages 5-8.

# Läkemedel för djur – en enkät

## Ditt behov och dina åsikter om läkemedelsanvändning till djur

Vi har tagit fram en enkät för att kartlägga veterinärers och djursjukvårdspersonals behov och åsikter relaterat till användning av läkemedel för djur, som ett led i Läkemedelsverkets arbete för att påskynda kommunikationen av ny säkerhetsinformation till användarna och öka biverkningsrapportering (inklusive brist på effekt).

Dina svar kommer att direkt påverka hur vi utvecklar information kring biverkningar och biverkningsrapportering.

Tack för att du deltar i undersökningen!

Undersökningen är anonym.

Om du vill få chansen att vinna en hållbar tygväska från Läkemedelsverket, vänligen ange din e-postadress.

### Del 1. Om mig

## 1. Ja tack, jag vill få chansen att vinna en hållbar tygväska från Läkemedelsverket! (förutsätter att du lämnar din e-post) [ text fält ]

## 2. Jag är...

- Veterinär
- Leg. djursjukskötare
- Djurskötare/Djursjukvårdare
- Veterinärstudent
- Student på Djursjukskötareprogrammet
- Annat [ text fält ]

## 3. Jag jobbar för närvarande...

- i en klinikverksamhet (huvudsakligen smådjur)
- i en klinikverksamhet (huvudsakligen lantbruksdjur)
- i en klinikverksamhet (huvudsakligen hästar)
- inom industrin
- på myndighet
- vid universitet/forskningsinstitution
- jag är student
- inte (arbetslös/pensionär)
- Annat [ text fält ]

### Del 2. Biverkningsrapportering

## 4. Jag har misstänkt eller märkt en biverkning eller brist på effekt i samband med läkemedel för djur...

- en gång per år
- 2 - 4 gånger per år
- 5 till 10 gånger per år
- mer än 10 gånger per år
- aldrig

## 5. Jag brukar rapportera...

- alla biverkningar/brist på effekt
- enbart oväntade biverkningar/brist på effekt
- enbart allvarliga biverkningar/brist på effekt
- aldrig

6. Jag brukar rapportera biverkningar eller brist på effekt via...

- Rapporteringsblanketten på Läkemedelsverkets webbplats
- Rapporteringsblanketten i eget journalsystem till Läkemedelsverket
- Direkt till Läkemedelsverket via telefon
- Direkt till företaget via telefon
- Direkt till företaget via e-post
- Vet ej
- Annat [ text fält ]

## 7. Jag skulle vilja ha möjlighet att rapportera biverkningar eller bristande effekt via... (ranka ditt svar, där 1 är ditt förstahandsval)

- Post
- Fax
- Telefon
- E-tjänst (direktrapportering via Läkemedelsverkets webbplats
- Via eget journalsystem
- E-post
- App i mobilen
- Annat

## 8. Om annat - ange gärna hur du skulle vilja ha möjlighet att rapportera biverkningar/brist på effekt här [ text fält ]

## 9. När jag har skickat in en biverkningsrapport, skulle jag uppskatta att få en återkoppling.

- Ja, via e-post vid varje rapport
- Ja, via e-post för oväntade/ allvarliga biverkningar / brist på effekt
- Ja, via en sammanställning av rapporter årsvis i en tidskrift t.ex. Svensk Veterinärtidning
- Nej, jag behöver ingen återkoppling
- Vet ej
- Annat [ text fält ]

## 10. Jag skulle oftare rapportera biverkningar eller brist på effekt om...

- Det fanns ett lätt och smidigt sätt att rapportera
- Jag fick återkoppling på min rapport
- Jag visste mer om hur min rapport hanteras och bidrar till förändringar
- Jag visste mer om vilka biverkningar som ska rapporteras
- Annat [ text fält ]

### Del 3. Tillgänglighet av ny säkerhetsinformation

## 11. Jag uppdaterar mig om nya ändringar av säkerhetsinformation t.ex. nya biverkningar, kontraindikationer och försiktighetsåtgärder….

- varje gång jag använder en produkt
- då och då
- vid uppdatering av information i journalsystem, inklusive råd till djurägare
- endast för nya produkter eller produkter som jag använder för första gången
- aldrig

## 12. Jag använder en eller flera av följande källor för att hitta ny säkerhetsinformation…

- Bipacksedeln
- Europeiska läkemedelsmyndigheten EMA:s webbplats
- Läkemedelsverkets webbplats
- FASS
- Google
- Företagets webbplats
- Letar aldrig efter ny säkerhetsinformation

## 13. Jag tycker att ny säkerhetsinformation (välj ditt mest relevanta svar):

- ska markeras tydligare i produktinformation inklusive bipacksedeln
- ska samlas på en lätt tillgänglig plattform/hemsida
- ska skickas till mig som ett brev/ epost
- ingen förändring behövs – är redan lätt att hitta
- annat [ text fält ]

## 14. Jag känner till Europeiska läkemedelsmyndighetens (EMA) dokument, med månatliga uppdateringar av säkerhetsinformationen avseende centralt godkända produkter (publ på engelska)…

- Ja och jag tycker att dokumentet är användbart
- Ja men jag tycker att dokumentet skulle vara mer användbart om det fanns på svenska.
- Ja men jag tycker inte att dokumentet är användbart.
- Nej

## 15. Jag känner till att ny säkerhetsinformation avseende centralt och nationellt godkända produkter publiceras på svenska i Läkemedelsverkets nyhetsbrev till veterinärer…

- Ja och jag tycker säkerhetsinformationen i nyhetsbrevet är användbar
- Ja, men jag tycker inte att säkerhetsinformationen i nyhetsbrevet är användbar
- Nej, inte alls
- Nej, jag använder en annan källa (fritext ruta) [ text fält ]

## 16. Kommentarer eller önskemål om förbättringar [text fält]

## Tack för din medverkan!

Resultatet av den här undersökningen planeras att publiceras i vårt nyhetsbrev för veterinärer

Rapportering av biverkningar (inklusive brist på effekt) är ett viktigt verktyg för att så snabbt som möjligt identifiera och därmed minimera riskerna för negativa läkemedelseffekter. Övervakning av rapporter sker kontinuerligt och kan leda till olika åtgärder t.ex. ändring i säkerhetsinformation.

Har du frågor, eller annat du vill diskutera är du välkommen att kontakta oss på Läkemedelsverket genom:

James Mount, veterinär, Enheten för läkemedelssäkerhet
[james.g.mount@lakemedelsverket.se](mailto:james.g.mount@lakemedelsverket.se)

Karin Sjöström, veterinär, Enheten för läkemedelssäkerhet
[karin.sjostrom@lakemedelsverket.se](mailto:karin.sjostrom@lakemedelsverket.se)

Läkemedelsverkets nyhetsbrev för veterinärer

Om du inte redan är prenumerant, är du välkommen att prenumerera på vårt nyhetsbrev för veterinärer: [lakemedelsverket.se/veterinarnytt](https://www.lakemedelsverket.se/veterinarnytt)

**Om behandling av e-postadresser**

De e-postadresser som lämnas i enkäten kommer endast behandlas av Läkemedelsverket. Personuppgifterna omfattar enbart e-postadresser och kommer från ifyllande av enkäten. Syftet med personuppgiftsbehandlingen är att kunna meddela vinnare av tygväska. Uppgifterna kommer sparas under den tid som enkäten pågår. Därefter raderas uppgifterna. Läkemedelsverket är personuppgiftsansvarig för behandlingen.

[Om GDPR och behandling av personuppgifter hos Läkemedelsverket](https://www.lakemedelsverket.se/sv/om-lakemedelsverket/administration/behandling-av-personuppgifter)

# Medicines for animals - a survey

## Your requirements and opinions about drug use in animals

We have developed a survey to collated the requirements and opinions of veterinary professionals related to the use of VMPs, which is part of the Swedish Medical Products Agency's work to accelerate the communication of new safety information to users and increase side adverse reporting (including lack of efficacy).

Your answers will directly impact developments in relation to communication and adverse event reporting.

Thank you for participating in the survey!

The survey is anonymous.

If you would like to get the chance to win a sustainable cloth bag from the Swedish Medical Products Agency, please enter your e-mail address.

### Part 1. About me

## 1. Yes, thank you, I want to get the chance to win a sustainable cloth bag from the Swedish Medical Products Agency! (leave your email)

## I am...

- Veterinarian
- Licensed veterinary nurse
- Animal caretaker
- Veterinary student
- Student on veterinary nursing program
- Other

## 3. I currently work ...

- in a clinic practice (mainly small animals)
- in a clinic practice (mainly farm animals)
- in a clinic practice (mainly horses)
- in industry
- at an authority
- at university / research institution
- a student
- unemployed / retired
- other [ text field ]

### Part 2. Adverse reaction reporting

## 4. I have suspected or noticed an adverse event or lack of efficacy following the use of a veterinary medical product ...

- once a year
- 2 - 4 times a year
- 5 to 10 times a year
- more than 10 times a year
- never

## 5. I usually report ...

- All adverse events / lack of efficacy
- Only unexpected adverse events / lack of efficacy
- Only serious adverse events / lack of efficacy
- Never

**6. I usually report adverse events or lack of efficacy via ...**

- Reporting form on the Swedish Medical Products Agency's website
- Reporting form in the own medical record system to the Swedish Medical Products Agency
- Directly to the Swedish Medical Products Agency by telephone
- Directly to the pharmaceutical company by phone
- Directly to the pharmaceutical company via e-mail
- Do not know
- Other [ text field ]

## 7. I would like to be able to report adverse events or lack of efficacy via ... (rank your answer, where 1 is your first choice)

- Post
- Fax
- Telephone
- Online form (direct reporting via the Swedish Medical Products Agency's website)
- Via own medical record system
- Email
- App via
- Other

## 8. If other - please indicate how you would like to be able to report adverse events / lack of efficacy [ text field ]

## 9. Once I have submitted an adverse event report, I would appreciate receiving feedback.

- Yes, by e-mail after each report
- Yes, via email for unexpected / serious adverse events / lack of efficacy
- Yes, via a compilation of reports annually in a publication e.g. Swedish Veterinary Journal
- No, I do not need any feedback
- Do not know
- Other [ text field ]

## 10. I would report adverse events or lack of efficacy more often if ...

- There was a quick and easy way to report
- I received feedback on my report
- I knew more about how my report contributes to changes
- I knew more about which adverse events to report
- Other [ text field ]

### Part 3. Availability of new product safety information

## 11. I update myself about new product safety information e.g. new adverse events, contraindications, and warnings….

- every time I use a product
- occasionally
- when updating information in medical record system, including advice to animal owners
- only for new products or products that I use for the first time
- never

## 12. I use one or more of the following sources to find new product safety information…

- Package leaflet
- European Medicines Agency EMA website
- Medical Products Agency's website
- FASS
- Google
- Pharmaceutical company website
- I never search for new product safety information

## 13. I think that new product safety information (choose your most relevant answer):

- should be marked more clearly in product information, including the package leaflet
- should be collected on an easily accessible platform / website
- should be sent to me as a letter / email
- no change needed - is already easy to find
- other [ text field ]

## 14. I am familiar with the European Medicines Agency (EMA) document, with monthly updates of the product safety information regarding centrally authorised products (published in English)…

- Yes and I think the document is useful.
- Yes but I think the document would be more useful if it was in Swedish.
- Yes but I do not think the document is useful.
- No.

## 15. I am aware that new product safety information regarding centrally, and nationally approved products is published in Swedish Medical Products Agency's newsletter to veterinarians…

- Yes and I think information in the newsletter is useful
- Yes, but I do not find the information in the newsletter useful
- No, not at all
- No, I use another source [ text field ]

## 16. Comments or requests for improvements [ text field ]

## Thank you for your participation!

The results of this study are planned to be published in our newsletter for veterinarians.

Reporting of adverse events (including lack of efficacy) is an important tool to identify risks as quickly as possible and thereby minimize the risks of adverse events. Monitoring of reports takes place continuously and can lead to various measures, e.g. changes to product safety information.

If you have questions, or anything else you want to discuss, you are welcome to contact us at the Swedish Medical Products Agency:

James Mount, Veterinarian, Department of Drug Safety
[james.g.mount@lakemedelsverket.se](mailto:james.g.mount@lakemedelsverket.se)

Karin Sjöström, Veterinarian, Department of Drug Safety
[karin.sjostrom@lakemedelsverket.se](mailto:karin.sjostrom@lakemedelsverket.se)

[Swedish Medical Products Agency's newsletter for veterinarians](https://www.lakemedelsinformation.se/sv/om-lakemedelsverket/press-och-nyheter/nyhetsbrev/nyhetsbrev-for-veterinarer#hmainbody2)

If you are not already a subscriber, you are welcome to subscribe to our newsletter for veterinarians: [lakemedelsverket.se/ veterinarnytt](https://translate.google.com/translate?hl=en&prev=_t&sl=sv&tl=en&u=https://www.lakemedelsverket.se/veterinarnytt)

**About handling of email addresses**

The e-mail addresses provided in the survey will only be handled by the Swedish Medical Products Agency. The personal information only includes e-mail addresses and comes from completing the survey. The purpose of personal data handling is to be able to contact winners of a cloth bag. The information will be saved during the time the survey is in progress, then the data will be deleted. The Swedish Medical Products Agency is responsible for handling of this personal data.

[About GDPR and processing of personal data by the Medical Products Agency](https://translate.google.com/translate?hl=en&prev=_t&sl=sv&tl=en&u=https://www.lakemedelsverket.se/sv/om-lakemedelsverket/administration/behandling-av-personuppgifter)
